# Editorial: Renewable chemistry

**DOI:** 10.3389/fchem.2025.1639782

**Published:** 2025-06-23

**Authors:** James Clark

**Affiliations:** University of York, York, United Kingdom

**Keywords:** renewable, biomass, green chemistry, renewable resources, carbon dioxide

The chemical manufacturing and allied industries are vital to the production of almost all articles in modern society, but they use largely well-established processes based on chemistry that is often over a hundred years old. As we move towards the use of (bio-) renewables, and with the drive towards a circular economy what chemistries will dominate future manufacturing processes and what chemicals will they manufacture? Increasing legislative pressure and growing user demands for low environmental footprints will add to the challenges but these are also opportunities for new chemistry and for new chemicals in the processes and as products. While green chemistry was originally established to create new, less wasteful and less hazardous processes, there is growing interest in creating new value chains for chemical products based on alternative (bio- or waste-based) resources and including the production of products compatible with a circular economy.

Frontiers Green and Sustainable Chemistry seeks to encourage the use of green processes using renewable resources either derived from sustainable biomass or from waste streams. This includes the green chemical or biochemical processes for the extraction from and conversions of biomass and chemically rich wastes including plastics and textiles. This area can be referred to as Renewable Chemistry.

In this Research Topic of Frontiers Green and Sustainable Chemistry “Renewable Chemistry” we see cutting edge examples of the production and utilisation of renewable resources which adhere with the principles of green chemistry by minimising the environmental footprints from the feedstock to the product. In Multi-step pre-treatment of rice husk for fractionation of components including silica, we see how one common form of biomass can become the feedstock to make a range of important products. By using a multi-step approach with all the steps involving low environmental technologies, we can make lignin, silica, and cellulose, all of which can be used as materials or as starting points for making important chemical products.

Carbon dioxide is most thought of as a pollutant that contributes towards climate change, but it is also a readily available C1 feedstock that could replace a significant proportion of traditional organic chemicals. In Effect of alkali metal cations on dehydrogenative coupling of formate anions to oxalate, we learn how to optimise the critical conversion process for turning CO_2_-derived formate into oxalates. In this way, we can make fully sustainable C2 chemicals that can in turn be converted in useful chemicals and polymers, thus relieving the demand for traditional petro-derived building block chemicals like ethene.

Aromatics are hugely important in modern chemistry and are a critical part of many useful materials as well as chemical products. While we have grown used to widely available, low-cost aromatics derived from petroleum, these are non-renewable and should be replaced. Fortunately, aromatics are abundant in nature including in tannins as well as in lignin. The challenge we face is to efficiently utilise these renewable resources to make valuable aromatic products. In Exploring tannin structures to enhance enzymatic polymerisation, we see how one of the leading green chemistry technologies, enzyme catalysis, can be used to convert low cost, abundant but also complex tannins into advanced materials. In particular, we learn about the effects of different components of tannins on the enzymatic polymerisation process. By tuning the process, we can make thermally stable materials with potential applications including the critical area of flame retardancy.

